# Lightweight saliency detection method for real-time localization of livestock meat bones

**DOI:** 10.1038/s41598-023-31551-6

**Published:** 2023-03-18

**Authors:** Tao Xu, Weishuo Zhao, Lei Cai, Xiaoli Shi, Xinfa Wang

**Affiliations:** 1 School of Artificial Intelligence, Henan Institute of Science and Technology, Xinxiang, 453003 China; 2 School of Information Engineering, Henan Institute of Science and Technology, Xinxiang, 453003 China

**Keywords:** Engineering, Optics and photonics

## Abstract

Existing salient object detection networks are large, have many parameters, are bulky and take up a lot of computational resources. Seriously hinder its application and promotion in boning robot. To solve this problem, this paper proposes a lightweight saliency detection algorithm for real-time localization of livestock meat bones. First, a lightweight feature extraction network based on multi-scale attention is constructed in the encoding stage. To ensure that more adequate salient object features are extracted with fewer parameters. Second, the fusion of jump connections is introduced in the decoding phase. Used to capture fine-grained semantics and coarse-grained semantics at full scale. Finally, we added a residual refinement module at the end of the backbone network. For optimizing salient target regions and boundaries. Experimental results on both publicly available datasets and self-made Pig leg X-ray (PLX) datasets show that. The proposed method is capable of ensuring first-class detection accuracy with 40 times less parameters than the conventional model. In the most challenging SOD dataset. The proposed algorithm in this paper achieves a value of Fωβ of 0.699. And the segmentation of livestock bones can be effectively performed on the homemade PLX dataset. Our model has a detection speed of 5fps on industrial control equipment.

## Introduction

Traditional pork boning is commonly done by hand. This results in low production efficiency, poor operational precision, and easy cross-contamination during production. It also takes up a lot of labor. In recent years, some large meat processing enterprises have partially adopted automated boning equipment. However, these devices cannot adapt themselves to the variability of livestock carcasses. This greatly affects the accuracy of the boning robot. The development of precision boning robots is a major boost to the livestock products processing industry. The precision boning robot relies on the vision module for precise identification and planning of the boning path. The segmentation of skeletal objects from X-ray images is an important prerequisite for subsequent path planning. Currently, the segmentation or classification of X-ray and CT images using deep learning methods is mostly used in the medical field. We realized that accurate segmentation of skeletal regions in pork X-ray pictures is of great importance for autonomous path planning and subsequent operation of the boning robot. According to our survey findings. The study of using saliency detection for X-ray image segmentation currently exists only in the medical field and is mostly used for the direct identification and classification of lesions. So far it has not ventured into the field of modern livestock meat processing. To this end we have drawn on many excellent studies on the application of neural networks^1–10^. These excellent research results come from a wide variety of research areas. Inspired by these methods and combined with the problems encountered in practical work. In this paper, we will start from the saliency detection method and pioneer the study of how to segment the bones in pork X-ray images. We also combine the actual working environment of the boning robot, so that the proposed model can run smoothly on an industrial control machine with limited hardware performance.

Saliency object detection(SOD) is a task that segments the regions or objects of greatest interest in human vision from the scene. It has a wide range of applications in many vision tasks. This includes image segmentation^11,12^, image retrieval^13^, object detection^14^, visual tracking^15^, image compression^16^, and scene classification^17^. Traditional approaches rely on manually designed underlying features and various heuristic a priori assumptions^18,19^. These methods lack advanced semantic information leading to unsatisfactory accuracy of the final detection results. In recent years due to the rapid development of Convolutional Neural Networks (CNN). Deep learning-based saliency detection methods have made a great leap in prediction accuracy^20–29^. However, the price for the improvement in accuracy is a larger network size and more computational effort. These advanced saliency detection methods often have large model volumes. It runs very slowly even on devices with high performance graphics cards. Therefore, the application scenarios of such models are extremely limited. It is difficult to function on robots, mobile devices and industrial equipment. Hardware performance is limited in these scenarios due to device size and stability requirements.

The SOD task requires both high-level semantic features and low-level granularity features to locate salient objects and their details, respectively. Multi-scale information is also needed to handle salient objects of different sizes in different scenes. Although some lightweight backbone networks such as MobileNets^30^ and ShuffleNets^31^ are now widely used in mobile devices. However, these existing lightweight networks usually have poor feature representation due to limited model depth. Direct application of these lightweight backbone networks in saliency detection tasks is difficult to achieve the desired accuracy^32^. Moreover, in most of the saliency detection tasks based on encoder-decoder architectures. The low-level features from shallow networks. It contains rich spatial information and can highlight the boundaries of salient targets. The high-level features come from the deep network. It is rich in semantic information, such as significant target location information. However, during the upsampling process, this information may be gradually diluted. In order to make full use of multi-scale features in decoding. Previous saliency detection methods have designed different kinds of feature fusion strategies^27–29^. These fusion strategies using nested dense connections enhance the final detection accuracy though. Nonetheless, the overly dense nested connection operations greatly increase the number of parameters and the computational load of the network. This leads to poor efficiency of model operations.

To address the above issues. In this paper, we propose a lightweight saliency detection network. Reducing the model size and increasing the model speed while taking into account the prediction accuracy. In order to solve the problem of under-expression of feature capabilities inherent to lightweight networks. Inspired by Liu et al.^25^, we use a custom lightweight encoder in the encoding stage. A multi-scale attention module is also introduced to fully extract salient features. By using attentional mechanisms to reinforce important features and suppress unimportant ones during the encoding phase. At the decoding stage. Inspired by Huang et al.^26^. We propose a lightweight, full-scale skip connection method. Used to fuse coarse and fine-grained semantic features. This is different from the side output fusion used by most SOD methods. Each decoder layer of the full-scale skip connection incorporates small-scale features from encoding and large-scale features from decoding. These features capture both fine-grained semantics and coarse-grained semantics at full scale. The extracted salient features are maximized to utilize without using an overly intensive fusion strategy. At the end of the network. We added an additional residual refinement part. Used to further optimize the predicted images generated by the backbone network. Make it more homogeneous inside with clearer borders.

In summary, the main contributions of this paper are:

1. In this paper, we propose an end-to-end lightweight saliency detection network. Extraction of salient features using multi-scale attention module. The full-scale skip connection module fuses coarse and fine-grained semantic information. The residual refinement module refines the final predicted image. This is also the first saliency detection network for livestock X-ray image segmentation within our knowledge.
2. The experimental results of the proposed model in this paper on six publicly available datasets show that: Compared with the traditional significance detection network. The proposed method in this paper has a smaller size, faster running speed and quite competitive prediction results.
3. Test results on a self-made PLX (Pork Leg X-ray) dataset showed that: The method proposed in this paper can segment the pork leg bone intact. And it can reach 5fps on industrial control devices.

## Related work

### Traditional saliency detection

Traditional saliency detection models use various prior knowledge and low-level features of the image for saliency detection^18,19^. Although these methods are faster to calculate. However, the lack of high-level semantic features leads to their limited expressive ability. With the rapid development of deep learning techniques in recent years, more and more CNN-based saliency detection models have been proposed. Some earlier CNN-based methods used several fully connected layers before making predictions on images^20,21^. Although these methods have greatly improved in prediction accuracy compared to traditional methods. However, the use of fully connected layers leads to the loss of spatial semantic information in the features. It causes the final prediction results to be relatively coarse. Since the fully connected network (FCN)^33^ has been proposed and applied to image segmentation. The impact on the subsequent development of saliency detection has been far-reaching. Hou et al.^24^ introduced a hopping layer structure and short links in the overall edge detector. The multi-scale features are sufficiently extracted. Nevertheless, this method does not make full use of the valid information in the context. It does not perform well in terms of salient object details. To solve this problem, Wang et al.^26^ designed a global recurrent localization network. Using contextual information to accurately locate salient objects through weighted response maps. However, the method pursues too much local fineness and does not perform as well in the overall effect of salient targets. In recent years, the prediction accuracy of traditional saliency detection networks has been improving. Qin et al.^27^ designed a prediction-refinement architecture. A hybrid structural loss function is also proposed to optimize the salient objective both structurally and on the boundary. Feng et al.^28^ proposed attentional feedback module and boundary enhancement loss to optimize the saliency detection results. Qin et al.^29^ proposed a residual module based on pooling operations. This allows capturing multi-scale contextual information from different sizes of receptive fields. These traditional saliency detection models continue to set new records for accuracy. However, the disadvantages of such models are also obvious: The excessive model size and high arithmetic power consumption make it difficult to be practically applied in many devices that need it.

### Lightweight saliency detection

Lightweight saliency detection models are a recent emerging research direction. The aim is to minimize the model size while maintaining a certain prediction accuracy in order to increase the running speed. Liu et al.^32^ proposed a method based on stereoscopic multiscale attention. Different scales of channel attention and spatial attention operations are performed at each encoding stage. Element-wise addition is used instead of channel dimension concatenation to reduce the number of parameters as much as possible. Li et al.^34^ proposed a saliency detection network for optical remote sensing image segmentation. A custom lightweight VGG-16^35^ network was utilized as the backbone. A correlation module is used to mine object location information in high-level semantic features to generate coarse salient maps. Subsequently, refinement sub-networks are built in the decoding process to gradually optimize the coarse salient map and finally generate the fine salient map. Gao et al.^36^ proposed an extremely lightweight saliency detection network based on practical application requirements. The number of parameters of this network model is only 100 k. Train from the beginning without using a pre-trained model. Almost the same effects as using pre-trained models can be achieved. In general, most of the existing lightweight saliency detection models are targeted at a specific application.

## Method

**Figure 1:**
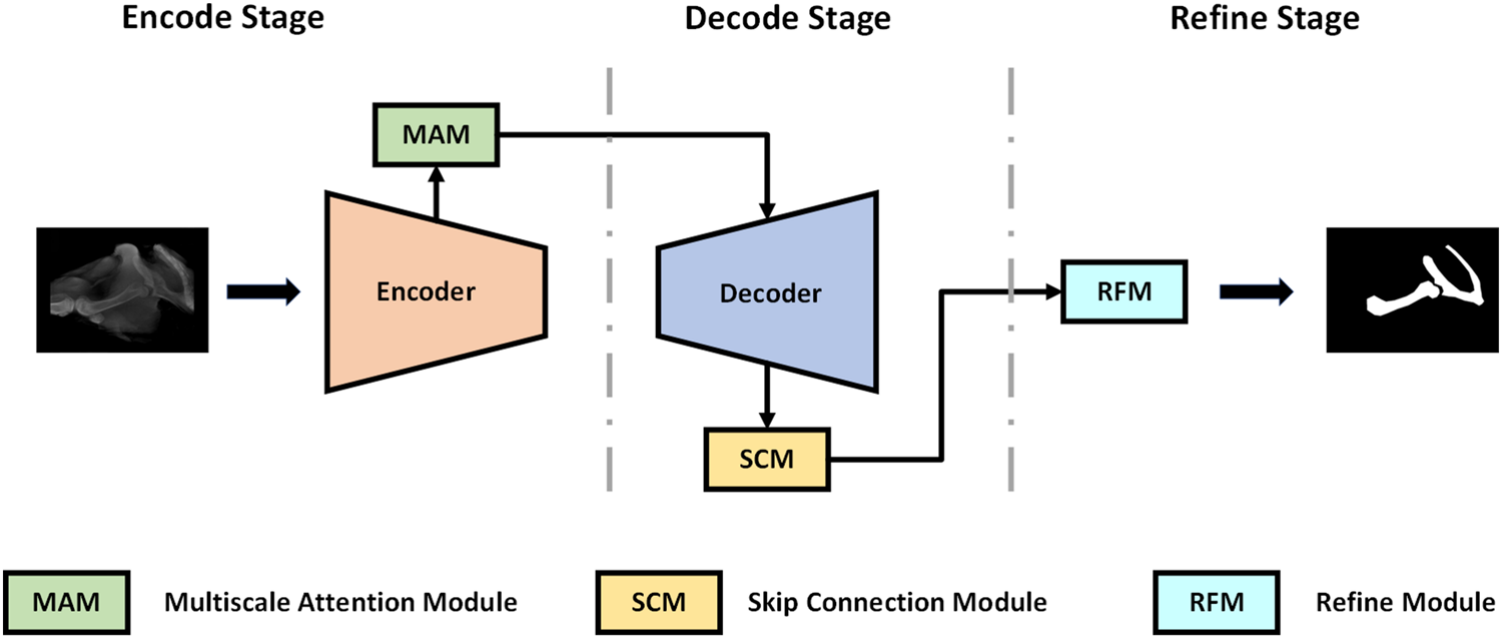
The general architecture of the proposed network.

In this section, we present the proposed SOD detection model in detail. As shown in Fig. 1, the model proposed in this paper is an encoder-decoder structure similar to the U-Net^37^ architecture. The image resolution of both input and output is 224 × 224. Unlike other SOD models, we do not use a large volume network as the backbone. In the encoder part, the feature extraction network of SAMNet^32^ is used. The entire network architecture consists of three parts: Encoder, decoder, and refinement part, respectively. The encoder part uses a multi-scale attention module to optimize the feature extraction results for each layer. The decoder part fully captures the semantic information using a fusion of jump connections. The refinement part uses a residual module to further refine the output of the decoder. Detailed information about the encoder, decoder, refinement part and the modules will be given in subsequent sections.

### Encoder

**Figure 2:**
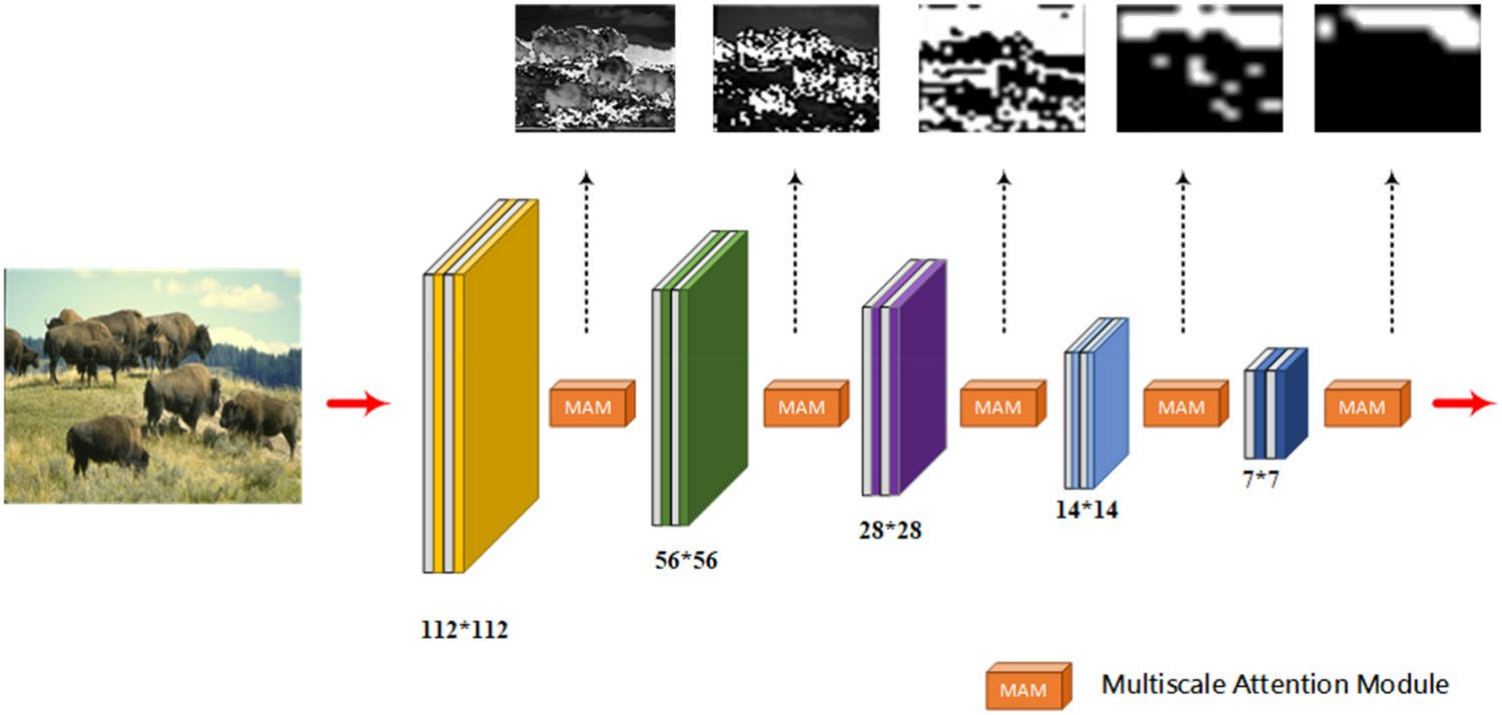
Architecture diagram of encoder.

The encoder part of the proposed model in this paper is a lightweight custom feature extraction network. As shown in Fig. 2. Our encoder network has five stages. Each stage consists of several convolution operations. Down-sampling and channel expansion operations are performed on the features at each encoding stage. Because traditional large volume feature extraction networks are not used. We control the number of channels of the feature in the encoder section. Only a limited number of channels are expanded in each coding phase, not exponentially as in other networks. After five stages of feature extraction operations, a feature map of 7 × 7 × 128 size is finally obtained.

**Figure 3:**
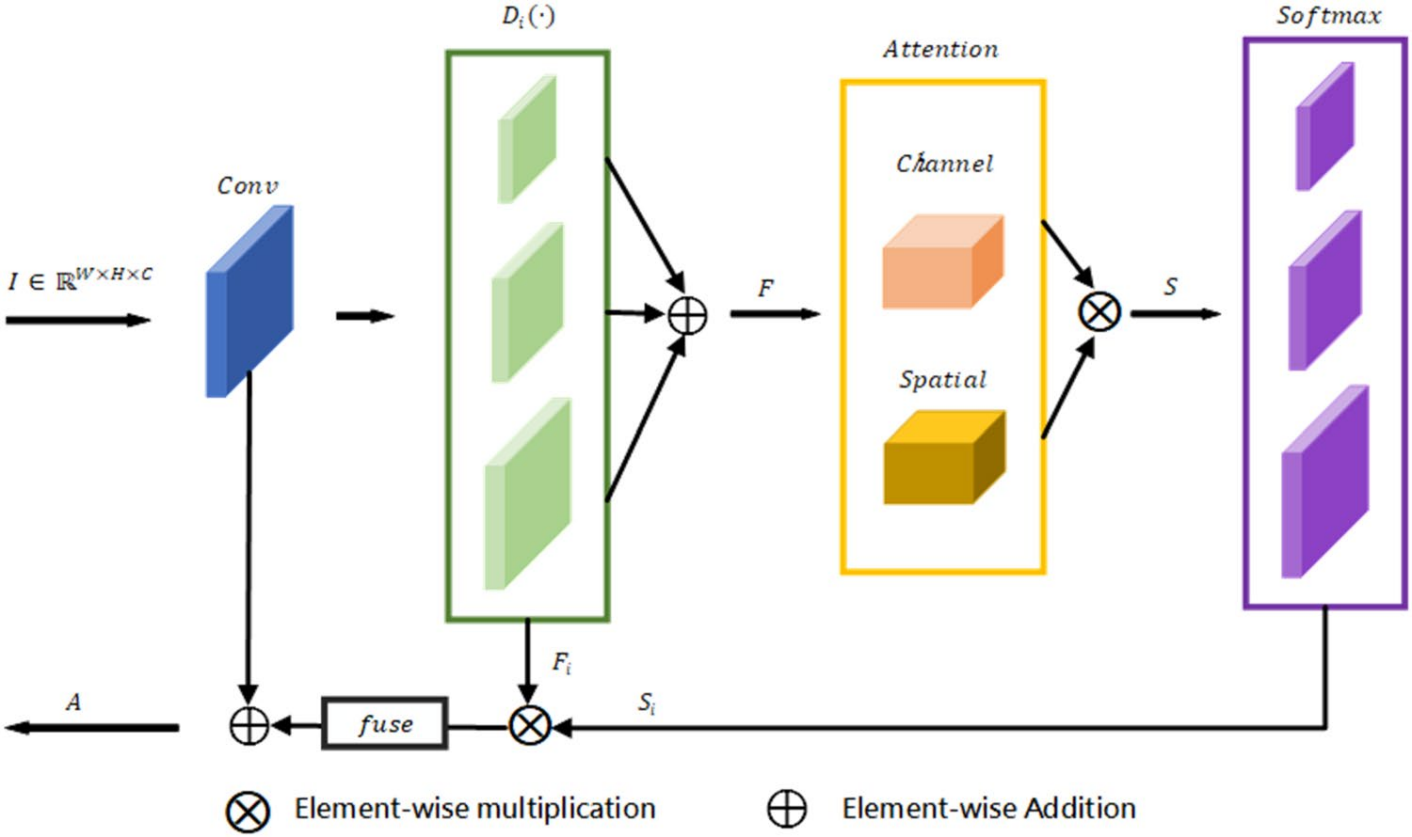
Multiscale attention module.

Unlike general encoders, the proposed encoder network in this paper introduces a multiscale attention module^32^ at each stage for feature optimization. Attentional mechanisms play a key role in human cognitive processes. Unlike a computer that can process an entire image at once, the human visual system filters relatively unimportant information such as background first. Channel attention can explicitly uncover the connection of features within a channel. And adaptively adjusts the feature images in a channel-by-channel manner. After channel-by-channel attention, some scholars introduced the concept of attention in space. Both channel attention and spatial attention belong to the category of self-attention. The spatial and channel self-attention can adaptively emphasize the most informative feature blocks and channels, respectively. The multiscale attention module in the model of this paper uses both attention mechanisms. This allows adaptive adjustment of the information flow in the different branches (see Fig. 3). Therefore, the multiscale attention module can extract as many effective features as possible in a lightweight network.

In the final part of each encoding stage, the feature maps are further processed using the multiscale attention module (MAM). Feature maps $I\in {\mathbb{R}}^{W\times H\times C}$. Where $W,H,C$ are the width, height, and number of channels, respectively. Extraction of its multiscale features using dilated convolution of different sizes:

1 $${F}_{i}={D}_{i}\left(Conv(I)\right) , \quad i=1, 2, 3$$

where $Conv(\cdot )$ denotes a set of convolution operations. It contains a regular convolution operation, a batch normalization operation, and a $ReLU$ nonlinear activation function. ${D}_{i}(\cdot )$ indicates different size of the dilated convolution operation.${F}_{i}$ is the multi-scale feature after processing. Here we use three different scales to expand the original feature map size. The three feature scales after processing are 2 times, 1 times and 0.5 times of the input features. After multi-scale processing. These feature maps allow the model to better cope with salient objects of different sizes.

Subsequently, the information at different scales is integrated using element-wise addition:

2 $$F={\sum }_{i=0}^{N}{F}_{i},$$

where $F$ is the integrated feature map. In order to reduce the computational overhead of the model, we use element-wise addition instead of the traditional concatenation operation for multi-scale information integration.

Subsequently, the integrated multiscale information is processed using two attention mechanisms:

3 $$S=Softmax\left(Channel\left(F\right) \otimes Spatial\left(F\right)\right),$$

where $Channel\left(\cdot \right)$ is the channel attention operation and $Spatial(\cdot )$ is the spatial attention operation. $\otimes$ s element-wise multiplication. $Softmax$ is the Softmax activation function。$S$ is the feature map after the attention mechanism calculation. Note that $S$ contains features on multiple scales. That is ${S}_{i} , i=\mathrm{1,2},3$. Channel attention operations and spatial attention operations are the two commonly used attention mechanisms. They reinforce the important features in the feature map in two separate ways. For channel attention, we first stretch the W, H dimensions in the input features ${F}^{W\times H\times C}$ into a one-dimensional vector, preserving the channel dimensions. The stretched features are then processed using a set of convolution operations with activation functions and the weights of each channel are obtained. The weights are then applied to the input features $F$. Similarly, for spatial attention, we first stretch the channel dimensions in the original input feature map into vectors. Then a set of convolution operations and activation functions are used to process and obtain the spatial weights, and finally the weights are applied to the original features.

After calculation, the final output of the MAM module is obtained as follows:

4 $$A=fuse\left({\sum }_{i=0}^{N}{F}_{i}\otimes {S}_{i}\right)\oplus I,$$

where $fuse$ is a multi − scale fusion operation. $A$ is the output feature map of the module containing the attention information.

### Decoder

The overall structure of the decoder network proposed in this paper is symmetrical to the encoder. The feature information from the encoder enters the decoder network after passing through a pyramid pooler. Most U-Net based models commonly use dense nested connections in different ways for feature fusion. This is to make better use of the extracted features in the decoding phase. However, this approach can greatly increase the number of parameters and the amount of computation. Inspired by UNet3 + ^38^, we designed a lightweight full-scale skip connection module (SCM). Each decoder layer contains smaller and same-scale feature maps from the encoder and larger-scale feature maps from the decoder. Thereby capturing both fine-grained and coarse-grained semantic information in its entirety.

For each decoding stage of the decoder the feature map ${X}_{de}^{i} , i=\mathrm{1,2},\mathrm{3,4}$. Similar to the U-Net network, the feature maps ${X}_{en}^{i}$ from the same number of layers in the encoding stage are first received directly. The difference is that skip connections are involved in fusion using more than just the same number of layers of features. It also needs to come with encoding features smaller than its own scale and decoding features larger than its own scale. These feature information are up-sampled, down-sampled and the number of channels modified respectively before fusion. The feature map ${X}_{de}^{i}$ is calculated as follows:

5 $${X}_{de}^{4}= P \oplus C\left(R\left({X}_{en}^{4}\right),R\left({X}_{en}^{3}\right),R\left({X}_{en}^{2}\right),R\left({X}_{en}^{1}\right)\right),$$

6 $${X}_{de}^{3}= {X}_{de}^{4} \oplus C\left(R\left(P\right),R\left({X}_{en}^{3}\right),R\left({X}_{en}^{2}\right),R\left({X}_{en}^{1}\right)\right),$$

7 $${X}_{de}^{2}= {X}_{de}^{3} \oplus C\left(R\left(P\right),R\left({X}_{de}^{4}\right),R\left({X}_{en}^{2}\right),R\left({X}_{en}^{1}\right)\right),$$

8 $${X}_{de}^{1}= {X}_{de}^{2} \oplus C\left(R\left(P\right),R\left({X}_{de}^{4}\right),R\left({X}_{de}^{3}\right),R\left({X}_{en}^{1}\right)\right),$$

where $P$ is the output feature of the pyramid pooler. $R\left(\cdot \right)$ is the corresponding adjustment operation. Adjust its size and number of channels according to different input sizes. $\oplus$ is the element − wise addition operation. $C\left(\cdot \right)$ is the connection operation of the channel dimension. Because the overall model needs to remain lightweight, we discarded the full channel connection. For the 4 feature maps involved in the fusion. We reduce their number of channels to 1/4 before performing the channel dimension connection operation. Avoid exponential expansion of the number of parameters at the time of fusion. To minimize the loss of accuracy caused by this change. At the end of each stage we make use of the idea of residuals. The result of the fusion is added by element-wise with the output of the previous stage.

### Refinement part

The proposed refinement module (RFM) in this paper optimizes the final output of the model by learning the residual between the prediction map ${M}_{coarse}$ and Ground Truth output from the decoder, ${M}_{residual}$.

**Figure 4:**
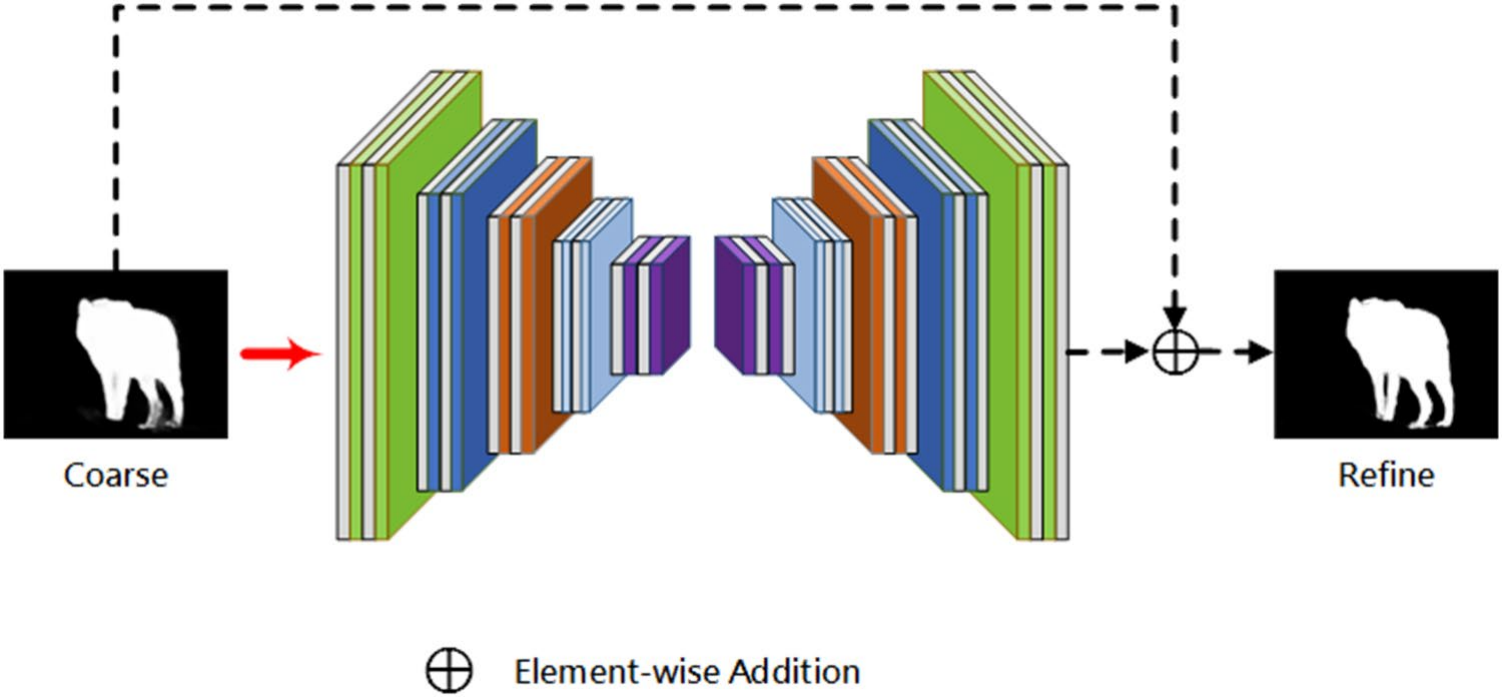
Residual optimizer.

To optimize the incomplete areas and blurred boundaries in the salient map. Inspired by Qin et al.^27^. We designed a lightweight residual optimizer. The optimizer uses a residual encoder-decoder architecture, which is shown in Fig. 4. Its main architecture is similar to our prediction module but simpler. It contains an encoder, a decoder and a residual output layer. Both the encoder and decoder have five stages. Each stage has only one layer convolution operation. In order, there are 4, 8, 16, 24, 36 filters of size 3 × 3, followed by a batch normalization operation and a ReLU activation function. The input and output are summed by element-wise at the end of the optimizer to output the final saliency map:

9 $${M}_{refine} = {M}_{coarse} \oplus {M}_{residual},$$

## Experiment

### Dataset

In this paper, the proposed model is evaluated on six commonly used benchmark dataset datasets for saliency detection. They are SOD^39^, ECSSD^40^, DUT-OMRON^41^, PASCAL-S^42^, HKU-IS^43^, DUTS^44^, respectively. DUTS is the largest saliency detection dataset available. It consists of two subsets, DUTS-TR and DUTS-TE. DUTS-TR contains 10,553 images and is commonly used for training models. 5019 images of DUTS-TE are used for testing. Both datasets contain complex scenarios and multiple target categories. The SOD contains 300 images, most of which contain multiple salient objects. Most of these images have low contrast and salient targets that overlap with the image boundaries. ECSSD contains 1000 images of complexly structured natural content. DUT-OMRON has 5168 images, each of which has a relatively complex background and contains one or two objects. PASCAL-S consists of 850 images with cluttered backgrounds and complex foregrounds. HKU-IS contains 4447 images. Most of the images have more than one connected or unconnected foreground object.

In addition, a self-made dataset of X-ray images of pig legs is proposed in this paper to satisfy the practical needs. It contains 500 x-ray images of different parts of the pig’s leg.

Following the practice of many outstanding saliency detection models in recent years. Our model is trained using the DUTS-TR dataset. Random flipping is used during training to improve the generalization of the model.

### Implementation details

The model proposed in this paper is implemented using the PyTorch framework. The hardware configuration of the server for training and testing is: Intel(R) Xeon(R) CPU E5-2630 v4 @ 2.20 GHz CPU, GeForce RTX 2080TI Graphics Cards, 32 GB RAM。The software is configured as: Ubuntu 18.04 Operating System, python3.8.10, PyTorch1.8.1, CUDA11.1. Follow the parameter settings used in many excellent saliency detection studies^32,45^. The model is trained using the Adam optimizer with an initial learning rate of 0.0003 and a cosine annealing learning rate adjustment strategy. The training epoch is 60. The number of model parameters is 2.1 M. The predicted speed is 5 FPS on an Industrial Personal Computer with a processor of i5-8750H.

### Evaluation metrics

In this paper, we use two metrics that are widely used in saliency detection to compare the precision of various methods. That is, the weighted F-measure value (${F}_{\beta }^{\omega }$), the mean absolute error (MAE). F-measure^46^ is a weighted summed average of precision and recall, and is a comprehensive evaluation method. It is calculated as follows:

10 $$F=\frac{(1+{\beta }^{2})\times \mathrm{Precision}\times \mathrm{Recall}}{{\beta }^{2}\times \mathrm{Precision}+\mathrm{Recall}},$$

where ${\beta }^{2}$ is generally set to 0.03, which puts more emphasis on precision. The weighted ${F}_{\beta }^{\omega }$ metric^47^ aims to correct interpolation flaws, dependence flaws, etc. in the traditional assessment metrics. We use it as one of the evaluation metrics. A larger value of ${F}_{\beta }^{\omega }$ represents a better accuracy of the result. MAE is the average of the difference of each pixel value between the predicted salient map and Ground Truth. It is calculated as follows:

11 $$MAE=\frac{1}{n}\sum_{i=1}^{n}\left|{y}^{i} - {p}^{i}\right|,$$

where ${y}^{i}$ is the pixel value of Ground Truth. ${p}^{i}$ is the pixel value of the predicted image. $n$ is the total number of image pixels. The smaller value of MAE means that the prediction result is closer to the true value and the better the algorithm works.

### Benchmark dataset performance analysis

In this paper, the proposed algorithm is compared with seven advanced and representative saliency detection methods. These include RFCN^48^, DSS^24^, PiCANet^49^, BASNet^27^, U2Net^29^ and two lightweight SOD methods HVPNet^45^ and SAMNet^32^. For fair comparison, all salient maps and parameter data for these methods were provided by the authors' papers or derived from their published code runs.

#### Quantitative comparison

In order to fully compare the proposed method in this paper with the existing models. Table 1 shows the detailed experimental results of the two selected metrics in this paper on the six benchmark datasets. The bottom half of Table 1 shows the lightweight saliency detection model proposed in this paper with two other recent lightweight detection models. It can be seen that the proposed method in this paper performs the best among the lightweight saliency detection methods. Compared to the state-of-the-art lightweight SOD model HVPNet and SAMNet on the selected six benchmark datasets. The results of both ${F}_{\beta }^{\omega }$ and $MAE$ metrics are all better than the two compared lightweight models. The top half of Table 1 shows some of the most representative methods of traditional SOD models in recent years. Comparing the data can be found. For the RFCN and DSS methods, the method in this paper achieves a comprehensive surpassing in accuracy. For the PiCANet method, the combined performance of this paper's method in the six data sets is almost on a par. For BASNet and U2Net, our method obtains a very competitive accuracy while using a very small number of parameters. For example, the average ${F}_{\beta }^{\omega }$ value of the best − performing model U2Net is 0.818, and the average ${F}_{\beta }^{\omega }$ value of the proposed method in this paper is 0.775. While the number of parameters of U2Net is 41.97 M, the number of parameters of our proposed method is 2.1 M. The accuracy of our method with a 20-fold reduction in the number of parameters is only 5% lower than that of U2Net.

**Table 1:** Comparison with five traditional methods and two lightweight methods on ${F}_{\beta }^{\omega }$↑ and $MAE$↓.

Method	Param (M)	SOD		ECSSD		PASCAL-S		HKU-IS		DUT-OMRON		DUTS-TE	
		Fωβ	MAE	Fωβ	MAE	Fωβ	MAE	Fωβ	MAE	Fωβ	MAE	Fωβ	MAE
RFCN	134.69	0.581	0.168	0.698	0.107	0.624	0.132	0.680	0.089	0.524	0.110	0.585	0.090
DSS	62.23	0.698	0.118	0.835	0.052	0.718	0.080	0.821	0.039	0.643	0.074	0.701	0.064
PiCANet	32.85	0.721	0.108	0.865	0.047	0.781	0.088	0.847	0.042	0.691	0.068	0.748	0.054
BASNet	87.06	0.728	0.114	0.903	0.037	0.792	0.076	0.889	0.032	0.750	0.056	0.802	0.047
U2Net	41.97	0.748	0.108	0.910	0.033	0.797	0.074	0.890	0.031	0.757	0.054	0.804	0.044
HVPNet	1.23	0.687	0.123	0.852	0.053	0.742	0.090	0.837	0.045	0.696	0.064	0.727	0.057
SAMNet	1.33	0.686	0.123	0.855	0.053	0.734	0.092	0.837	0.045	0.699	0.065	0.729	0.058
Ours	2.1	0.699	0.122	0.864	0.050	0.752	0.089	0.854	0.041	0.726	0.061	0.754	0.054

**Figure 5:**
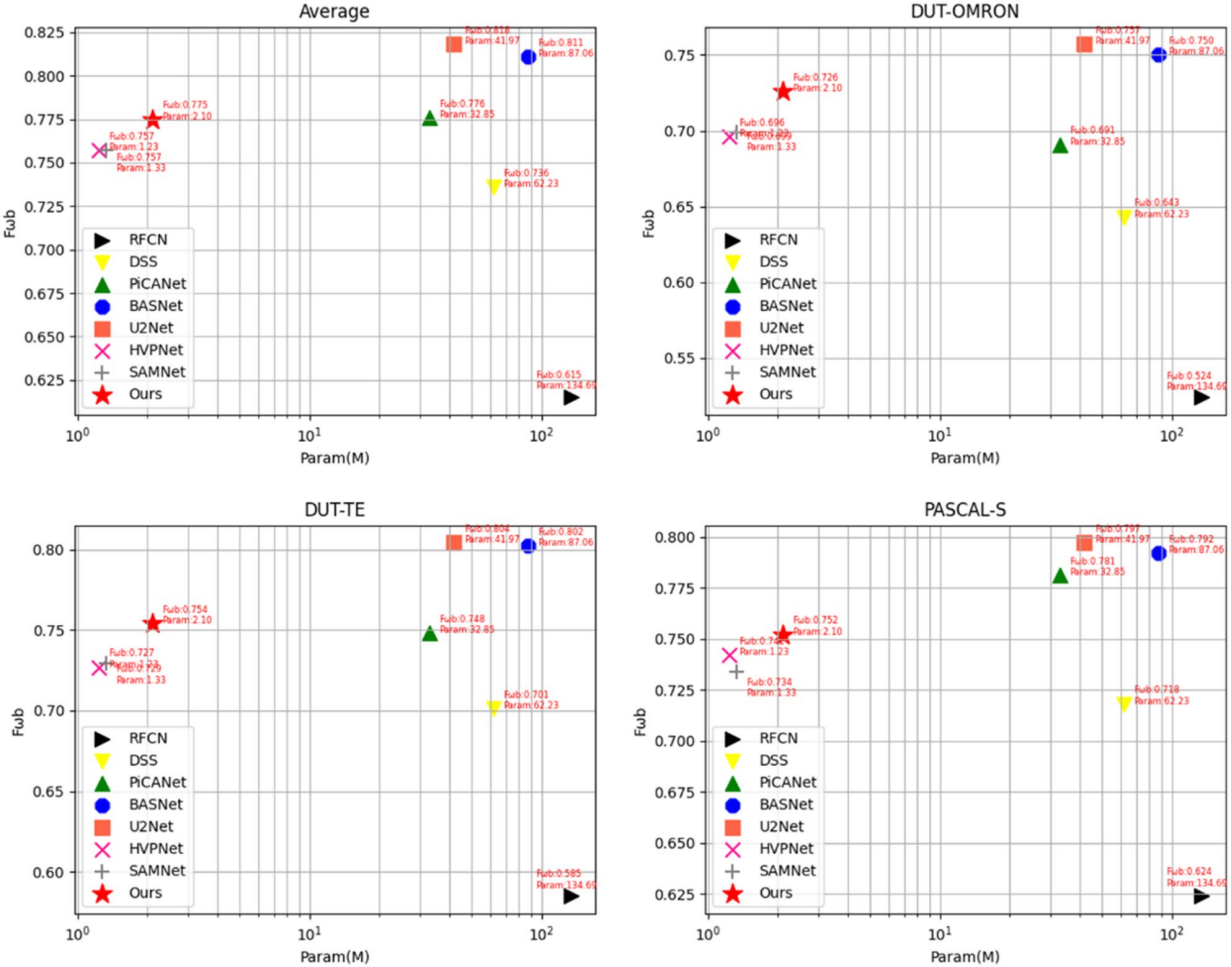
The number of model parameters and the metrics trade-off chart.

Figure 5 clearly shows the trade-off between the number of model parameters and the measured metric values. As shown in Fig. 5, we compared the three datasets DUT-OMRON, DUT-TE, and PASCAL-S, and the means on all datasets, respectively. Where the horizontal coordinate is the number of model parameters and the vertical coordinate is the value of ${F}_{\beta }^{\omega }$. The method proposed in this paper appears in the upper left corner in all comparison figures. This means that the proposed method in this paper achieves the accuracy of a state-of-the-art model with a much smaller number of parameters compared to the conventional SOD method. Compared with the lightweight SOD method, our method improves the accuracy of the model considerably without losing model speed.

#### Qualitative comparison

**Figure 6:**
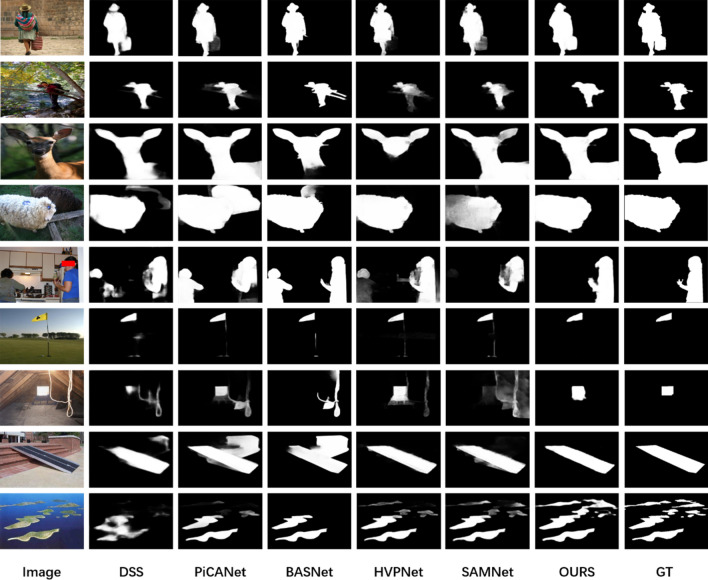
Qualitative comparison of the proposed method with the state of the arts SOD method.

To further demonstrate the effectiveness of the proposed method in this paper. In Fig. 6, we provide some visual examples to show the performance of the model. Although the lightweight method proposed in this paper is slightly inferior to the traditional SOD method with a large number of parameters in terms of measurement metrics. But it can still segment salient objects and their boundaries in many challenging scenes. Examples include complex scenes (rows 2 and 5), disorienting backgrounds (rows 1 and 6), large objects (rows 3 and 4), low contrast between foreground and background (rows 7 and 8), and salient objects that are not continuous (row 9).

As shown in Fig. 6. In rows 1 and 2, all methods roughly segment the contours of the salient objects, and only the method proposed in this paper is closest to the ground truth. In rows 3 and 4, the proposed method in this paper almost perfectly segmented the salient objects, while the other methods all had different degrees of deficiencies. In row 5, some methods incorrectly split the figure on the left side into salient objects due to the complex scene. There are also methods that do not completely segment the people on the right side of the picture. In row 6, only our method is unaffected by the flagpole and splits the flag accurately. In rows 7, 8, most methods do not correctly identify the salient regions due to the low contrast between foreground and background. Our method was hardly affected. In row 9, the salient objects are discontinuously distributed throughout the picture area. All methods segment the lower half of the island, but ignore the upper half. Only the method of this paper completely segmented the upper part of the island.

### X-ray dataSet performance analysis

To verify the effectiveness of the proposed method on the X-ray images of pig legs in this paper. We used the model proposed in this paper and MobileNetV2^50^ and MobileNetV3^51^ for separate training and testing against the pig leg X-ray images, respectively. The three models were trained for the same number of rounds following the criteria in the implementation details section.

**Figure 7:**
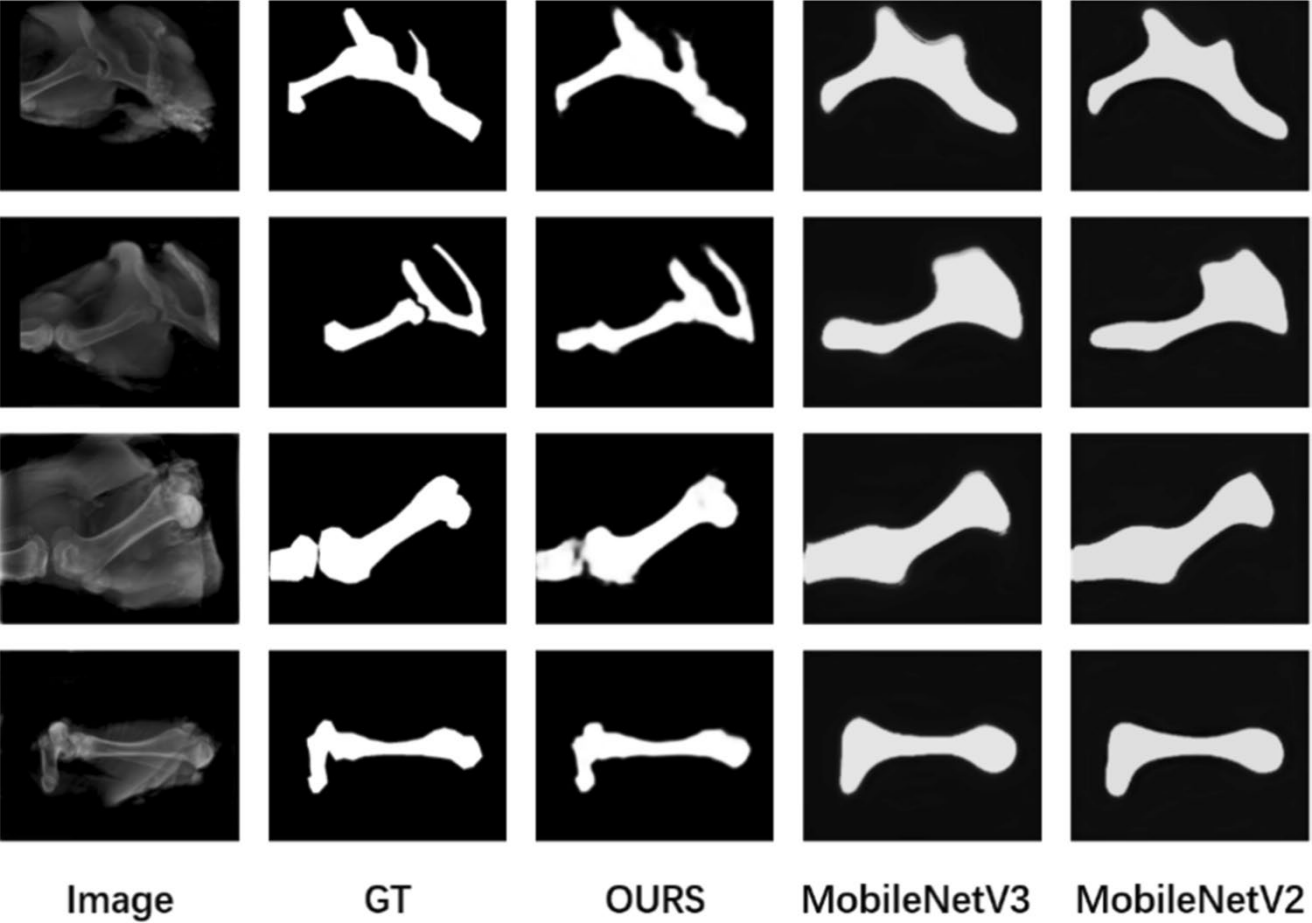
Qualitative comparison of the proposed method with a generic lightweight method on X-ray images.

As shown in Fig. 7, we selected images of four different locations of the pig leg to compare the qualitative effects of several methods. It can be seen that in all four images, the proposed model in this paper effectively segmented the skeletal regions. The skeletal regions segmented by the other two comparison methods contained a lot of confusing meat tissue.

### Ablation experiments

To verify the effectiveness of the proposed various modules, Table 2 shows the results of the ablation experiments for all the modules proposed in this paper. In order to compare all modules fairly, the experiments are based on the complete model of this paper and ablate the proposed modules separately. The comparison of the two measures was performed on the ECSSD dataset. It can be seen that the value of Fωβ decreased by 0.009 after ablation of MAM. The value of MAE increased by 0.003. Subsequent ablation experiments on the SCM and RFM modules, respectively, showed that the values of Fωβ decreased to different degrees and the values of MAE increased to different degrees. In summary, after ablating several modules separately. The values of both selected measures degenerate to different degrees. However, these degradations are very minor, which is a side-effect of the considerable degree of robustness of the infrastructure of the proposed model in this paper. In summary, the MAM, SCM and RFM modules proposed in this paper can all effectively improve the prediction quality of the model.

**Table 2:** Fωβ↑ and MAE↓ values for ablation experiments on the ECSSD dataset.

Ablation module	Fωβ	MAE
None	0.864	0.050
MAM	0.855	0.053
SCM	0.846	0.056
RFM	0.857	0.055

In summary. On the generic saliency detection dataset. The proposed lightweight saliency detection method in this paper achieves almost comparable prediction accuracy to traditional large volume models using a very small number of parameters. Our model achieves the best prediction accuracy compared to other lightweight saliency detection methods. On the X-ray dataset, the proposed method in this paper can segment the skeletal regions more accurately and effectively compared with other lightweight networks. It is almost unaffected by the meat tissue in the image. In terms of model volume and operating speed, our model has only 2.1 m of parameters and achieves comparable detection accuracy with tens of times less volume than conventional models. The detection accuracy is optimal compared to other lightweight models. In terms of practical applications, our model can run smoothly at 5pfs on the industrial control machine of the boning robot.

## Conclusion

In this paper, we do not only consider accuracy for SOD tasks. We also want to balance accuracy and running speed with the lightweight of the model. In this paper, a lightweight saliency detection network for real-time localization of livestock meat bones is proposed. The use of self-attentive mechanisms in the encoding phase allows the model to efficiently extract high-level features and low-level details. The use of lightweight skip connection in the decoding stage helps capture both fine-grained semantic and coarse-grained semantic information at full scale. Finally, this paper proposes a residual optimization module to optimize the significant region with respect to the boundary. Experimental results on six publicly available datasets show that. The method proposed in this paper is still comparable to the accuracy of state-of-the-art models with a small number of parameters. Experimental results on a self-made PLX dataset show that. Our method can effectively segment the skeletal regions in X-ray images. For an input image of 224 × 224 size. The proposed method achieves 5FPS on an Industrial Personal Computer with processor i5-8750H. The actual needs of the boning robot path planning are met in terms of speed and accuracy. And the model is highly scalable. It can be migrated and used on all tasks that require saliency detection as pre-processing, especially for hardware-constrained scenarios. In the future, we plan to further reduce the number of model parameters to better segment the skeletal regions only for X-ray images.

## Data Availability

The datasets used and/or analysed during the current study available from the corresponding author on reasonable request.
